# Editorial: Acute leukemias: molecular characterization, leukemia-initiating cells, and influence of the microenvironment

**DOI:** 10.3389/fonc.2023.1199354

**Published:** 2023-05-10

**Authors:** Garrett M. Dancik, Lokman Varisli, Ioannis F. Voutsas, Spiros Vlahopoulos

**Affiliations:** ^1^ Department of Computer Science, Eastern Connecticut State University, Willimantic, CT, United States; ^2^ Cancer Research Center, Dicle University, Diyarbakir, Türkiye; ^3^ Department of Molecular Biology and Genetic, Faculty of Science, Dicle University, Diyarbakir, Türkiye; ^4^ Cancer Immunology and Immunotherapy Center, Cancer Research Center, Saint Savas Cancer Hospital, Athens, Greece; ^5^ First Pediatric Clinic, University of Athens, Athens, Greece

**Keywords:** leukemia (acute myeloid), leukemia-initiating cells (LICs), microenvironment, cellular transition dynamics, cell death, drug resistance, biomarker

Acute leukemias remain a challenge, in spite of improvements in diagnosis and treatment. Fully establishing the depth and extent of the relative impact of the molecular mechanisms of disease progression and the pathways to recurrent disease will require decades of research, until our comprehension enables the routine development of simple and effective cures. This special issue was aimed to address progress in the characterization of the molecular basis of acute leukemias and to explore potential links with disease course. The articles presented in this issue deal with the subject in general from two main perspectives.

The first perspective in this Research Topic is the identification of factors that associate with a negative disease course, and potentially affect drug efficacy. Wang et al., investigated the effect of Nrf level on chemotherapy success in adult B-ALL patients and demonstrated a direct negative relationship between Nrf level and vincristine therapy response, for the first time. Mechanistically, they have uncovered that vincristine treatment causes an increase in the Nrf2 mRNA and protein levels in adult B- ALL patients, elevated Nrf2 decreases pro-apoptotic BAD protein level in a PI3K/AKT dependent manner and consequently decreases vincristine chemotherapy sensitivity. We report a retrospective study of pediatric ALL patients performed by Feng et al., on the adverse effect of CDKN2A/B deletions on clinical outcomes in pediatric B-ALL patients. Since the reports about the clinical effect of CDKN2A/B deletions in pediatric ALL are controversial, Feng et al. have conducted a comprehensive analysis of CDKN2A/B deletions in 599 pediatric B-ALL patients and consequently demonstrated that CDKN2A/B deletions were associated with older age at diagnosis, higher white blood cell counts, and prominent hepatosplenomegaly. Moreover, they also demonstrated that the patients with CDKN2A/B deletions had the worst clinical outcomes, especially in the TP53 deletion carriers. Zhang et al., establish a transcriptome-wide m7G methylome profile for AML and identified N7-methylguanosine modification of messenger RNA as a potential source of acute myeloid leukemia (AML) drug resistance. They showed that there were significant differences in the genes modified by m7G methylation in resistant AML cells, and the number of methylated genes and peaks in drug-resistant cells was greater than those in non-resistant cells. Zhang et al., explained why in moderate-to-severe lower respiratory tract infections, lung microbiome changes in children with hematological malignancies are a source of negative disease course. Hong et al., investigated for the probable correlations of TIM-3 expression between leukemic blasts and T lymphocytes since TIM-3 is thought to be involved both in the self-renewal of leukemic stem cells and the immune escape of AML cells. They demonstrated that TIM-3 expression levels of leukemic blasts and T lymphocytes in the bone marrow of *de novo* AML patients correlate with the presence of core binding factor (CBF) translocations, without having a detectable impact on the clinical outcome. Zhao et al. investigated the function of ECHDC3, which is upregulated in CD34+ progenitor cells of AML after chemotherapy, but whose prognostic significance and function are as yet unknown. They demonstrated that ECHDC3 alters the bone marrow microenvironment by inducing changes in the composition of infiltrating immune cells. Furthermore, they also showed that knocking down ECHDC3 in AML cells by RNAi promoted the death of leukemia cells with cytarabine and doxorubicin. Nilius-Eliliwi et al., demonstrate the power of optical genome mapping (OGM) to yield novel clinically significant results, including information helpful in disease monitoring and AML disease biology. They applied a “next-generation diagnostic workup” strategy with OGM and whole-exome sequencing (WES), and detected a DDX3X: MLLT10 gene fusion, which would otherwise be missed by routine diagnostics. They also discovered several aspects of lineage ambiguity, not shown by standard diagnostics: these included deletions of SUZ12 and ARPP21, as well as T-cell receptor recombination. Their findings could explain an aggressive disease course. Schwarz et al., characterized a method to inhibit the constitutive activity of oncogenic receptor FMS-like tyrosine kinase 3 internal tandem duplication (FLT3ITD) in AML cells, by disrupting oligomerization of receptor protein tyrosine phosphatase PTPRJ. They showed that interfering with PTPRJ self-association may be used as a tool to restrict oncogenic FLT3 activity, affecting FLT3-driven cell proliferation and clonal growth. Yuan et al., presented a hypothesis that germline mutation in RecQ-like helicase can be involved in hereditary predisposition to acute leukemias. They performed whole exome sequencing in peripheral blood mononuclear cells in a familial leukemia case, and for the first time to our knowledge, found a germline RECQL mutation potentially involved in hereditary predisposition to acute leukemia. The hypothetical biological process in which RECQL gene mutation (rs146924988) affects DSBs repair and mediates the generation of fusion genes provides a new understanding for the pathogenesis of leukemia, and highlights the necessity for next-generation sequencing-based screening of genes involved in this process in potential HPS patients. Xu et al., used weighted gene co-expression network analysis, extracting gene co-expression modules to relate them to clinical features, and identified TRIM32 as a potential driver of unfavorable AML disease course. Consequently, they demonstrated that TRIM32 is highly expressed in AML cells compared with cells from healthy donors and Knockdown of TRIM32 significantly inhibited the proliferation of AML cell lines, *in vitro*. Jiang et al., investigated the role of mitochondria-related metabolic reprogramming in the occurrence, development, drug resistance, and recurrence of acute myeloid leukemia. They analyzed immune infiltration and immunosuppressive genes and demonstrated that the mitochondria-related gene risk signature of AML patients was strikingly positively correlated with an immune cell infiltration and expression of critical immune checkpoints, indicating that poor prognosis might be attributed to an immunosuppressive tumor microenvironment. Takeda et al., presented a case of repeated lineage switches in acute leukemia and they reported that dynamic lineage conversion from ALL to AML occurred after clofarabine monotherapy was provided as a fourth induction regimen. They proposed that a monoallelic deletion and a frameshift mutation in TP53 gene accompanied by MLL gene amplification may have contributed to lineage plasticity and therapeutic resistance in this case, the first reported case of acute leukemia presenting with lineage ambiguity and MLL gene amplification.

The second perspective in this Research Topic concerns AML cell death, and generally, pathways to block the uncontrolled proliferation of acute leukemia disease clones. Zhong et al., investigated molecular patterns related to ferroptosis in AML cells. They integrated the genome information of 992 AML specimens, including expression profile chip data and high-throughput sequencing data, and analyzed the characteristics of immune cell infiltration, lipid metabolism, and inflammation development in the tumor microenvironment of AML patients. They used RSL3, a targeted inhibitor of GPX4, in AML cell lines HL-60 and THP1, to explore the biological effects of inducing ferroptosis. They demonstrated that RSL3 induced cell death in a dose-dependent fashion, indicating that GPX4 is a potential target for AML treatment. Li et al., constructed a cuproptosis-related lncRNA signature, to classify AML patients into high and low risk and revealed multiple signaling pathways, especially immune-related processes, were found to be significantly enriched in the high-risk group. In addition, their results indicated for AML patients in the high-risk group had a lower sensitivity to a range of anti-leukemia agents, including cytarabine, methotrexate, etoposide, and ABT-263 (a BCL-2 inhibitor, also called Navitoclax), while indicating a higher sensitivity to drugs like rapamycin, bortezomib, Erlotinib, even though some of them are currently not in clinical use for the treatment of AML. Tomaz et al., noted a significant induction of apoptotic AML cell death after treatment with the non-steroidal anti-inflammatory drug nimesulide, either alone or in combination with prednisolone. They also demonstrated that nimesulide potentiates the cytotoxic *in vitro* effect of several chemotherapy drugs used in AML, including cytarabine. They report the case of a patient with AML who presented a partial response after utilization of nimesulide, characterized by complete clearance of peripheral blood blasts and an 82% decrease of bone marrow blasts associated with myeloblast differentiation. Weighted correlation network analysis of serial whole-transcriptome data of cell lines treated with nimesulide revealed that the sets of genes upregulated after treatment with nimesulide were enriched for genes associated with autophagy and apoptosis, and on the other hand, the sets of downregulated genes were associated with cell cycle and RNA splicing.

We also received 2 comprehensive review articles that discuss the established knowledge with current literature. Yin et al., presented a survey of novel biomarker candidates, and their critical comparison to representative existing biomarkers. They concluded that integrated analysis of AML biomarkers from cytogenetics, molecular biology, and pathophysiology is conducive to the correct evaluation of prognostic factors to achieve the precise implementation of individualized hierarchical treatment. Gu et al., discussed the roles of Cancer-associated fibroblasts (CAFs) in acute leukemia. Although, CAFs can originate from diverse cell types, leukemia transformation is rare in patients with non-fibrotic myeloproliferative neoplasms. However, it is common in patients with myelofibrosis, which is also associated with an unfavorable disease course. Consequently, they concluded that CAFs in bone marrow may correlate with myelofibrosis, promote leukemia progression, and induce chemoresistance.

As the guest editors for the Research Topic “*Acute Leukemias: Molecular Characterization, Leukemia-Initiating Cells, and Influence of the Microenvironment* “ we thank all contributing authors and hope you enjoy these interesting papers.

## Author contributions

All authors listed have made a substantial, direct, and intellectual contribution to the work and approved it for publication.

